# Supplementary material for: Multiple memories can be simultaneously reactivated during sleep as effectively as a single memory
Source: Commun Biol. 2021 Jan 4;4:25. doi: 10.1038/s42003-020-01512-0 (PMC7782847; doi:10.1038/s42003-020-01512-0)
Supplement: Supplementary file 2 — Supplementary Information [file 42003_2020_1512_MOESM2_ESM.pdf]

Title: **Multiple memories can be simultaneously reactivated during sleep as effectively as a single memory**

Eitan Schechtman, James W. Antony, Anna Lampe, Brianna J. Wilson, Kenneth A. Norman & Ken A. Paller

Supplementary Materials include:

1. Supplementary Figure 1
2. Supplementary Figure 2
3. Supplementary Figure 3
4. Supplementary Table 1

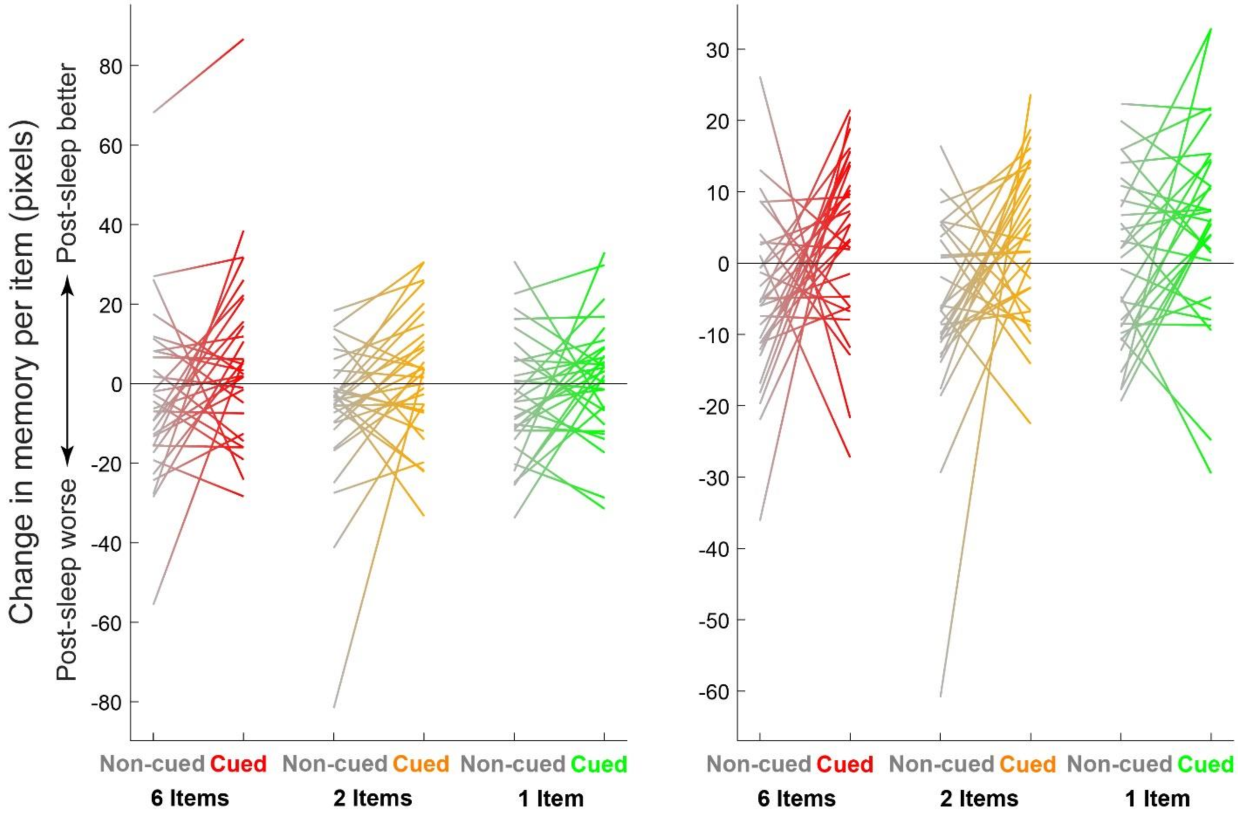

<u>Supplementary Figure 1</u>: **Individual participant averages of the change in accuracy over sleep for cued and non-cued items of different set sizes.** Results are shown for the uncorrected values used for the analysis shown in Figure 2 (left) and for the corrected values (in which the pre-sleep error rates were regressed out) used for supplementary analysis (right). Source data are provided as a Source Data file.

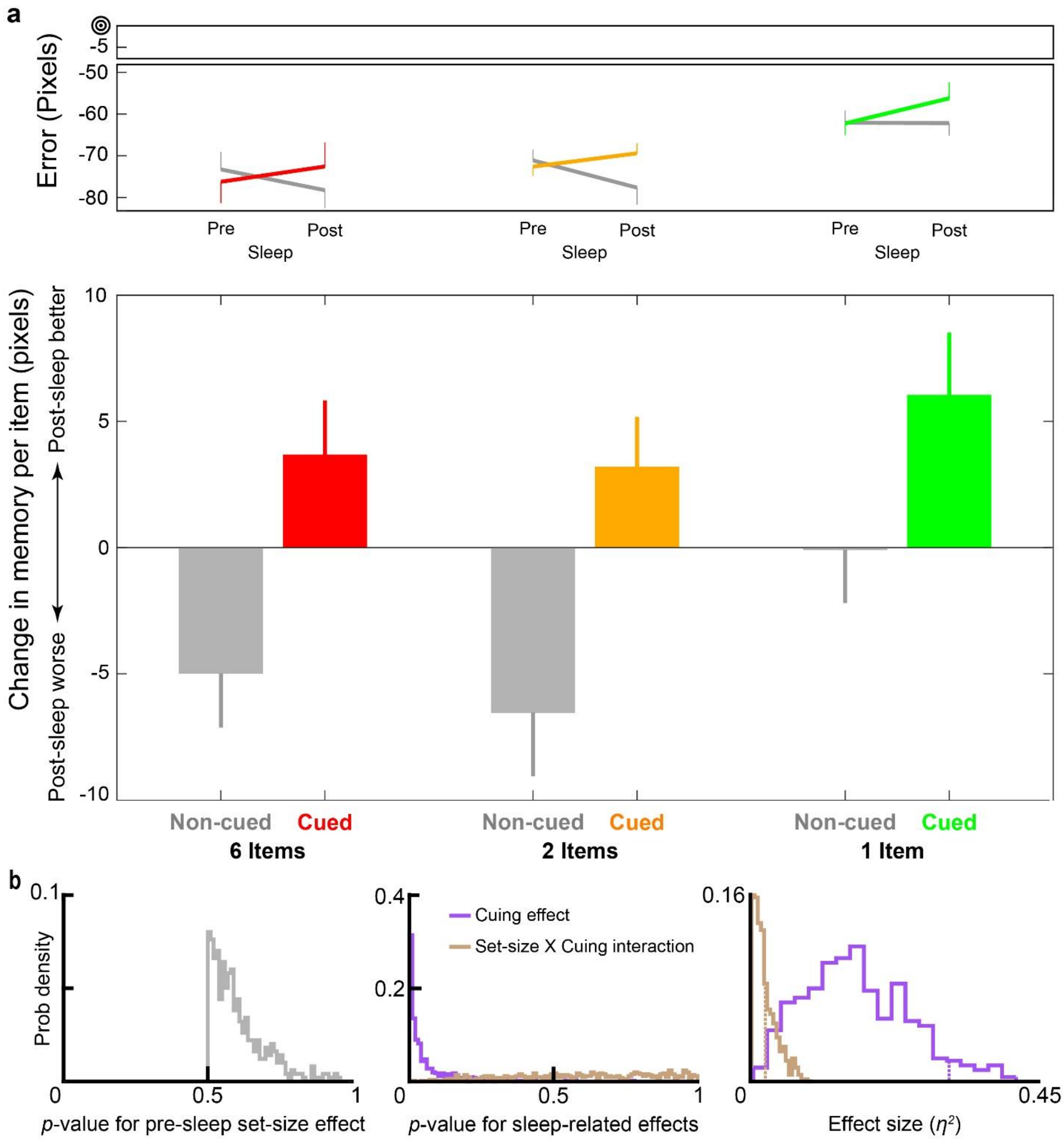

Supplementary Figure 2: **The effect of cuing and the null interaction effect between cuing and set-size are not a result of pre-sleep differences in errors between set-sizes.** (a) To rule this option out, we regressed the pre-sleep error rates out. Note that pre-sleep values are therefore identical to those presented in Figure 2a. As for the uncorrected data, with the corrected data the cuing effect is significant (p<0.001) and the cuing-by-set-size interaction is not (p=0.75). (b) To complement this

method, we subsampled data in a way that would minimize effects of pre-sleep differences (i.e., we chose a subsampled dataset that had a pre-sleep set-size difference with $p>0.5$, as shown in the histogram on the left). We then calculated *p*-values for the cuing effect and the cuing-set-size interaction for these datasets (histograms shown in center) and showed that the cuing effect was consistently significant whereas the interaction was consistently not. The right panel shows a histogram of the effect sizes (η^2) of the cuing effect (purple) and the interaction effect (brown) for the subsampled data sets. Dashed vertical lines show the effect sizes calculated for the full datasets (i.e., without subsampling). Source data are provided as a Source Data file.

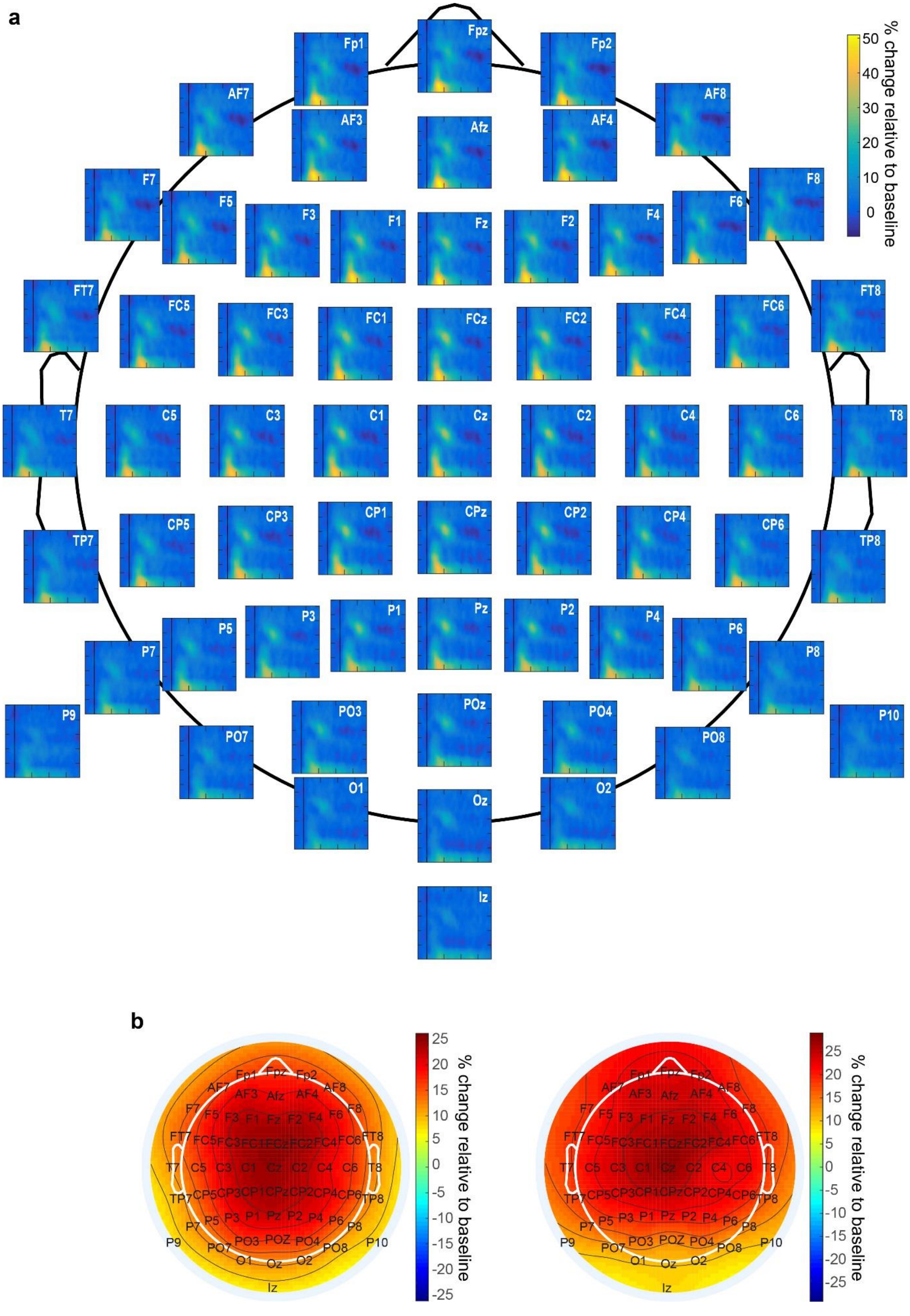
a
Fp1
Fpz
Fp2
AF7
AF3
Afz
AF4
AF8
F7
F5
F3
F1
Fz
F2
F4
F6
F8
FT7
FC5
FC3
FC1
FCz
FC2
FC4
FC6
FT8
T7
C5
C3
C1
Cz
C2
C4
C6
T8
TP7
CP5
CP3
CP1
CPz
CP2
CP4
CP6
TP8
P9
P7
P5
P3
P1
Pz
P2
P4
P6
P8
P10
PO7
PO3
POz
PO4
PO8
O1
Oz
O2
Iz
% change relative to baseline
50
40
30
20
10
0
b
% change relative to baseline
25
20
15
10
5
0
-5
-10
-15
-20
-25

Supplementary Figure 3: Additional physiological data. (a) Spectrograms for all scalp electrodes, locked to cue onset during sleep and averaged over trials regardless of condition. Image positions on the scalp reflect electrode positions. (b) Scalp distributions of power modulations for delta-theta (left) and sigma (right), the two main clusters shown in Figure 4b. Source data are provided as a Source Data file.

<u>Supplementary Table 1</u>: Themes used for sets of images

Theme	Used as
Balls	Two- or six-item sets
Fire	Two- or six-item sets
Birds	Two- or six-item sets
Documents and Literature	Two- or six-item sets
Cameras	Two- or six-item sets
Automobiles	Two- or six-item sets
Cats	Two- or six-item sets
Timepieces	Two- or six-item sets
Clothes with zippers	Two- or six-item sets
Dogs	Two- or six-item sets
Doors	Two- or six-item sets
Drinks	Two- or six-item sets
Food	Two- or six-item sets
Frogs	Two- or six-item sets
Heart	Two- or six-item sets
Kettles	Two- or six-item sets
Toilets	Two- or six-item sets
Trains	Two- or six-item sets
Phones	Two- or six-item sets
Musical Keyboard	Two- or six-item sets
Airplane	Two- or six-item sets
Cough	Two- or six-item sets
Flowers	Two- or six-item sets
Shoes	Two- or six-item sets
Pen	One-item sets
Trumpet	One-item sets
Violin	One-item sets
Monkeys	One-item sets
Kiss	One-item sets
Pen	One-item sets
Cow	One-item sets
Pig	One-item sets
Record	One-item sets
Money	One-item sets
Laugh	One-item sets
Toothbrush	One-item sets
Robot	One-item sets
Owl	One-item sets
Gong	One-item sets
Lobby bell	One-item sets
Drop	One-item sets
Boiling water	One-item sets
Slinky	Practice sets
Balloon	Practice sets
Computer keyboard	Practice sets
